# Evidence-informed stakeholder consultations to promote rights-based approaches for children with disabilities

**DOI:** 10.3389/fresc.2024.1322191

**Published:** 2024-04-29

**Authors:** Keiko Shikako, Jonathan Lai, Paul Y. Yoo, Gail Teachman, Annette Majnemer

**Affiliations:** ^1^ School of Physical and Occupational Therapy, McGill University, Montreal, QC, Canada; ^2^ Autism Alliance of Canada and Institute of Health Policy, Management and Evaluation (IHPME), Dalla Lana School of Public Health, University of Toronto, Toronto, ON, Canada; ^3^ The Hospital for Sick Children, Toronto, ON, Canada; ^4^ School of Occupational Therapy, Western University, London, ON, Canada

**Keywords:** human rights, disability rights, childhood disability, knowledge translation, health, policy, participatory research

## Abstract

**Purpose:**

To strengthen the translation of evidence to actionable policy, stakeholder engagement is necessary to synthesize, prioritize and contextualize the academic research content into accessible language. In this manuscript we describe a multi-level evidence-based stakeholder consultation process and related outcomes proposed to promote awareness of and foster cross-sectorial collaborations towards human rights-based approaches for children with disabilities.

**Methods:**

Mixed-methods participatory action research done in three steps: (1) A literature review of peer-reviewed evidence on rights-based approaches in childhood disabilities; (2) Consultation with researchers in diverse fields, grassroot organizations, caregivers, and youth with disabilities; (3) A constructive dialogue with decision makers at federal and provincial levels in Canada to discuss consultations results.

**Results:**

Stakeholders value human rights approaches that can have a direct impact on practical aspects of their daily living. Organizations give high importance to adopting rights-based approaches to measure policy outcomes, while parents value service provision and youth emphasize accessibility.

**Conclusion:**

The implementation of rights-based approaches in childhood disabilities can support policy, services, and daily lives of children with disabilities and the ecosystems around them. It can also guide research priorities, and create a common language to foster collaborations across sectors and interested parties.

## Introduction

Children with disabilities are no longer considered objects of protection but rather as subjects of rights. However, at the intersection of rights for persons with disabilities and rights for children, they are a minority within a minority (1–3). Two international treaties address the rights of children with disabilities: The United Nations Convention on the Rights of the Child (CRC) and the Convention on the Rights of Persons with Disabilities (CRPD) (4, 5). The CRC ensures the rights of children with four guiding principles: non-discrimination; best interest of the child; the right to life, survival and development; and the right to participate. It was the first human rights treaty to include protection against discrimination based on disability. The more recent CRPD enshrines the rights of individuals with disabilities to live in dignity, with equal rights and opportunities.

Signatory countries to both conventions are obliged to have an active national reporting system, as part of the implementation process of these treaties. However, in Canada, the implementation of the CRPD has been deficient, as evidenced in the United Nations' 2017 *Concluding Observations on the Initial Report of Canada*. In the report, the UN CRPD committee stated concerns about the absence of data collected on children with disabilities, the lack of formal consultations on comprehensive plans for the implementation of the Convention, and the absence of information on mechanisms to foster the participation of children with disabilities in consultations. The committee also reported on the lack of established criteria for applying the principle of the best interests of the child. These concerns involve key prerequisites for full implementation of the Convention and further realization of rights of Canadian children with disabilities. This is significant since a rights-based approach to the provision of services could emphasize and bring into view opportunities for continuous supports for children with disabilities. A focus on the best interests of the child can lead to the creation of health, education and social services that respond to their needs, allocating resources to the most pressing issues indicated by children and families, valuing their priorities, and raising awareness about persistent rights violations (3).

It follows that both research and policy in this domain should be informed by voices of children with disabilities and their families to elucidate their interests. Research impacts are amplified when end-users are involved from the beginning of the research process (6, 7). Early citizen engagement informs research priorities and defines uptake strategies that are relevant in the current political context and adapted to the current system's capacity (e.g., infrastructure, technical and human resources) (8, 9). The relevance and fit of uptake strategies are important given that influencing policy is often a desired research outcome. However, most research findings for children of disabilities do not influence policy decisions even though the findings may be relevant (10–12). Opportunities for positive influences are missed with this disconnect between research evidence and policy even as the research-informed policymaking and its importance has been well established (13). Multiple barriers maintain the gap between policy and evidence (14). In particular, limited “availability and access to research/improved dissemination” as well as a lack of “clarity/relevance/reliability of research findings” diminish research influence on policy (15).

In policy contexts, reliance on passive or unidirectional knowledge translation renders most research outputs ineffective (16). This is because, in contrast to the linear process of the scientific method, policy processes are typically nonlinear. Even specific research to policy strategies, which are rare, tend to overlook the contextual factors—such as values and personal experience, political will and pragmatism—that affect a policy maker's decision-making (17, 18). Hence, it is crucial to “frame” evidence and key strategies in line with what matters to policymakers and use their language to tell engaging “stories” that animate evidence with stakeholder input and priorities (19, 20).

Co-production of knowledge in health research with stakeholders generates relevant and useful evidence for decision making (21). It is beneficial and potent to include a broad range of stakeholder groups who can contribute unique perspectives, skills, and resources; more so when the knowledge synthesized aims to inform policy (20). In rehabilitation research, advances have been made toward involving adults with disabilities and their caregivers (20). Yet, other groups such as policymakers, community organizations, and children and youth with disabilities are less often represented (22). Here, we report on the process and results of including stakeholders' voices to inform actionable strategies from research literature concerning the structures, processes, and outcomes in the implementation of rights-based approaches in policy development in Canada. We performed a literature review of peer-reviewed evidence and presented the results to grassroot organizations, caregivers and youth with disabilities. These groups were consulted to rate the importance and relevance of the evidence. The input received from the stakeholders' consultation was then presented to key decision makers in the federal service, in hopes of informing policy in the realm of child disability. This paper recounts the findings from the stakeholder consultation.

## Methods

**Figure 1:**
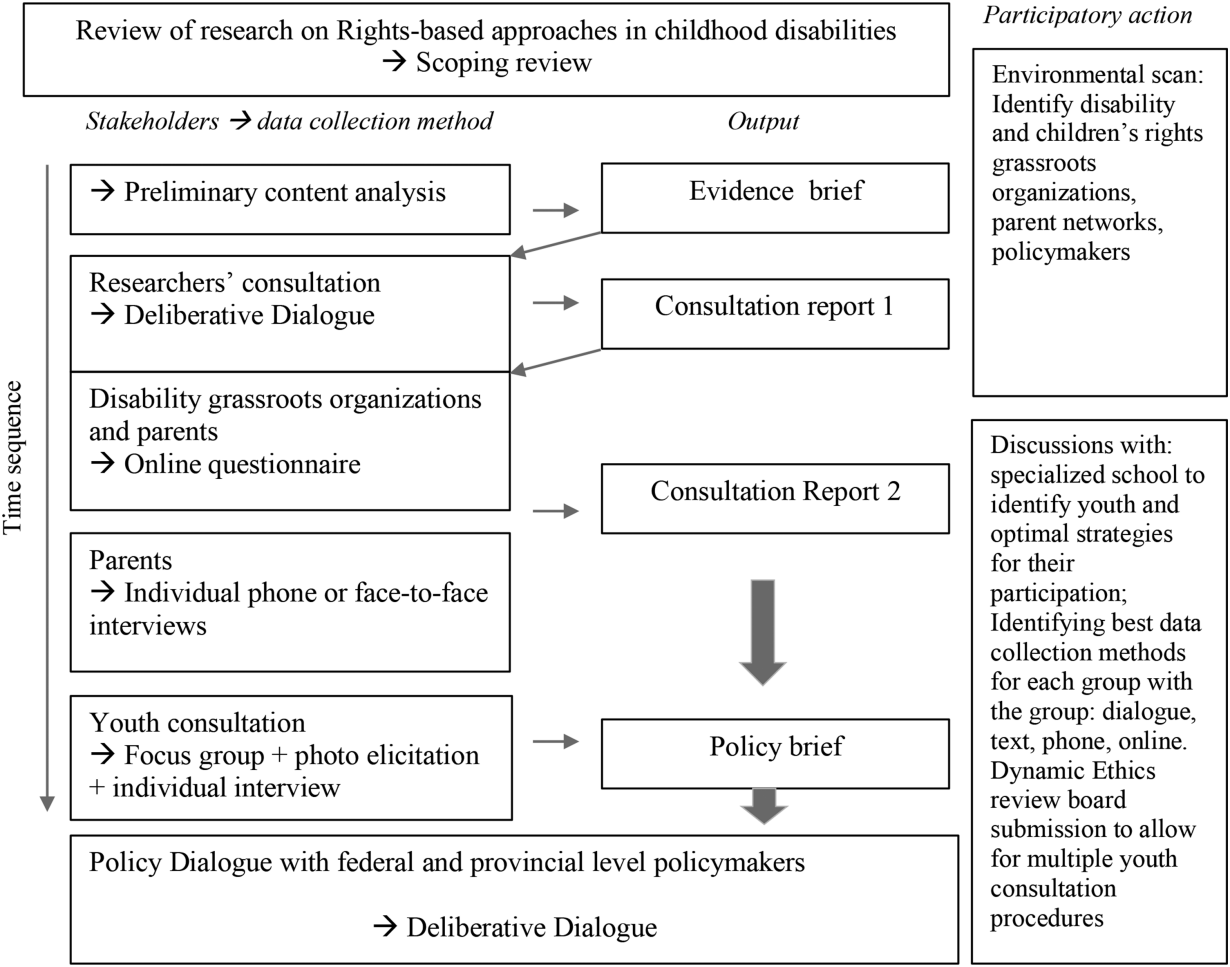
Stakeholder consultation steps and data collection sequence.

This mixed-methods study employed a Participatory Action Research approach to validate the priorities identified in a research review of rights-based approaches in childhood disabilities by key stakeholders in the childhood disabilities ecosystem in Canada. A sequential data collection procedure was adopted with one step informing the next (23, 24). In the extended period of time given for data collection, elements of the scoping review and the multiple consultations informed the next steps of data collection and analysis. The qualitative elements (i.e., open ended answers to questions in an online survey, e.g., “how this issue impacts your organization clientele or your child?”, interviews and stakeholder dialogues) informed the analysis of the quantitative data [i.e., Likert scale questions in an online survey, e.g., objective ratings (1–5) of importance of issues presented], and were also used to provide in-depth understandings of the descriptive quantitative data (e.g., sociodemographic information, sum of importance score by stakeholder group). We do consider the qualitative elements to be the core of the project, with the supplemental component being the quantitative analysis. The stakeholder consultation steps and data collection sequence are illustrated in Figure 1.

### Preliminary evidence review in preparation for stakeholder consultations

A systematic search on current evidence on rights-based approaches for children with disabilities was conducted. Searches yielded a total of 1,754 results and 174 articles were included after systematic screening. Content analysis was performed with the data extracted from the search. The content analysis of the research articles revealed four major themes: inclusion, participation, self-advocacy and equity. The themes were then further probed to frame the issues connected to the themes and to identify proposed solutions. The issues and solutions identified under each theme were classified according to messages of interest to different key stakeholder groups, including: families, grassroots/community organizations, children, health care providers, policy makers and researchers. We then identified the articles from the UN CRC and the CRPD that related to the issues presented to better frame solutions within a rights-based approach. Table 1 illustrates a sample of how we framed the questions within the themes from the review and aligned them with specific CRC and CRPD articles.

**Table 1:** Qualitative analysis guide for data generated with youth in the study.

Guiding analytic questions	Sub-questions	Specific data probes
What are the conditions that mediate disabled children's opportunities to access their most basic human rights?	What are the conditions of participants’ everyday lives (material conditions, activities and routines, social contexts—as described in their accounts and as illustrated in their photos)	Do the youths’ accounts and their photographs illustrate ways that families of children and youth with disabilities might experience a higher burden of care, financially and socially?
Which mediators are aligned with the intersection of childhood and disability?	How do youth participants and/or their photos identify “barriers and supports” or mediators that influence their participation	Examine the data to consider youths’ participation in: (a) community activities, (b) decision-making, discussions and actions that impact them directly and (c) accessing social resources and supportive services

Three products, for three different stakeholder groups were generated from the results of the scoping review to inform the current study: (1) a research brief (for researchers); (2) an online questionnaire listing issues related to the implementation of rights-based approaches in childhood disabilities and proposed solutions identified in the research literature (for disability organizations and parents of children with disabilities); (3) guiding questions for interviews (for youth with disabilities). Procedures for application of each product are described below.

### Participants and data collection procedures

#### Researchers

Using convenience sampling, we recruited 15 interdisciplinary researchers in the field of childhood disabilities and human rights (expertise included humans rights and law, philosophy, critical disability studies, social work, knowledge translation, health and social policy, rehabilitation science and ethics). The group participated in a 3-h in-person deliberative dialogue on the subject of rights-based approaches for children with disabilities in the Canadian context (25). Prior to the dialogue, participants were provided with the evidence brief; the brief summarized the issues identified under each of the themes, and proposed evidence-based solutions on how society at large, different systems of care, and government can cooperate to integrate rights into policy.

The deliberative dialogue was facilitated by a moderator who proposed a semi-structured sequence of topics for discussion with a request that participants contribute to each topic within the brief. First, participants were asked to comment on the topic they perceived as most critical. Then, they were asked to examine key issues, plausible solutions and key implementation considerations for that topic. They were also asked to consider what could be relevant for other stakeholder groups (i.e., families, children and youth, educators, policymakers). Participants discussed a range of approaches (e.g., rights-based, civic model) for engagement, and strategies for maximizing knowledge translation, optimizing breadth in stakeholder consultations, and tailoring communications with each stakeholder audience. The dialogue was audio-recorded to augment the detailed notes taken by two members of the research team. A summary of the dialogue was sent to participants after 2 weeks, and they were asked to provide feedback if any point was not clear or did not convey the ideas that had been discussed. Analysis of salient topics, key messages to different stakeholder groups, and points for knowledge translation strategies were used to guide a secondary analysis of the literature review results (with a focus on services, key messages for each stakeholder group), and to further refine the online stakeholder questionnaires and focus group questions.

#### Grassroots organizations

We consulted with grassroots organizations using an electronic questionnaire [completed online on RedCap (Research Electronic Data Capture)]. Grassroots organizations (and parents as described in the section that follows) were queried about six overarching issues as identified in the scoping review and centred around the themes of inclusion, participation and caregiver support. Respondents rated the importance of each issue to the mandate of their organization, rating it from 1 to 5 (1—not important, 5—very important). For each issue, 4–10 actionable solutions were listed (also from the research literature review). These solutions for each issue were also rated on the same scale of 1–5, identifying how important each solution was to address that specific issue.

A list of non-governmental disability organizations was generated in collaboration with governmental and non-governmental offices, and through organizations listed in a mobile App (Jooay—a free mobile App listing adapted and inclusive leisure programs across Canada), and by manual searching on different hubs of Canadian disability groups. Organizations recruited were Canadian Civil Society Organizations (CSOs) and Disability Persons Organizations (DPOs) that work with government in matters related to the rights of persons with disabilities and/or children (an initial list was provided by the Canadian Office for Disability Issues), foundations that offer services and supports for children and families with disabilities, and community organizations offering leisure and community participation opportunities for children with disabilities. Further participating organizations were recruited through snowball sampling where each organization invited was asked to nominate others that might be interested. Key informants were identified and invited by email to serve as, or help identify, a respondent for their organization. Participants could save and continue completing the questionnaire in multiple rounds, with reminders sent every 2 weeks, prompting completion over a 6-month period.

#### Families and caregivers of children with disabilities

A convenience sample of families of children with disabilities were recruited online through social media and parent support groups. Parents were invited to complete an online questionnaire via RedCap that mirrored questions posed to stakeholder organizations, but modified to align with a parent focus (e.g., “to what extent is this issue important for my organization” was modified to read “to what extent is this issue important for my child”). The format of the consultation was modified iteratively to include an option for individualized face-to-face discussions when some parent participants indicated that the “human rights” topic was complex and not a common language used by parents. Accordingly, individual interviews, using the online questionnaire as a point-by-point guide (see Questionnaire Design section below), were offered to all parent participants; this allowed researchers to explain each question, and provided space for parents to provide rich detail in their responses.

#### Youth with disabilities

A purposive convenience sample of youth/young adults with disabilities were recruited from the senior class of a school for children with disabilities in Montreal, Canada. Specifically, students in the classroom had a primary physical disability, communication challenges, and medical conditions. This group encompasses a large spectrum of developmental disabilities (e.g., cerebral palsy, autism, rare chromosomic conditions). Once a week, they attended a regular secondary school, within a classroom only for students with disabilities. Although the convenience sample did not include all disability groups, we hope this sample can shine light into a group of youth that is rarely represented in research and advocacy, frequently having their basic human rights violated, according to both conventions (CRC and CRPD) (26). Potential participants were introduced to the project and invited to participate through an activity led by the research team in their school classroom. The activity introduced the topic of human rights, the rights of persons with disabilities and the rights of children, as well as the UN Convention and potential implications of these in daily life.

Drawing on interview and photo-elicitation methods developed in earlier work by Teachman and Gibson (27), youth who consented to participate were provided with access to cameras over a 2-week period as co-researchers who generated photos to help show and discuss their perceptions of how their human rights were being respected (or not) in different activities and moments of their lives (27). Photo cameras or iPads were installed on their wheelchair, with appropriate accessibility to switch-activated devices as needed and tested with the support of a technician from the rehabilitation centre. The first photo try-outs were done with two members of the research team during an occupational therapy session in the school. During this session, the research team members explained the procedures to obtain consent from other people who may be in the photos they wanted to take, presenting a “consent to take image” slip. Consent slips, including a short explanation of the project, were provided to all participants with instructions to show the slip to people who may be in their photos. Sample photos were taken to ascertain that participants knew how to manipulate their devices and compose photos for the research.

Individual follow-up interviews, structured by discussion of each participant's photos (28, 29), were scheduled 2–3 weeks following the camera loan and at times convenient to participants (30). For example, as the research and participant reviewed the participant's photos, the researcher asked probing questions such as: Tell me about this photo? Why did you choose to take this photo? What do you think this photo shows about whether or not your human rights are being respected? How would you want to change this situation to improve recognition of your human rights? (31) Participants were supported to choose their preferred interview method; some elected to use email with back-and-forth responses between the youth and the team member while others preferred to participate in a face-to-face interview at school or at home, with or without their parent's or educator's mediation to facilitate communication, and/or using communication devices. With consent, interviews were recorded and transcribed. A paper audit and data trail were kept for all interactions. Field notes summarizing conversations involving multiple communication modes [parents' clarification of verbal responses from their child-participant, responses mediated by augmentative and alternative communication (AAC) devices, interviewer's clarifications and interpretation of participants' accounts], transcriptions, and written communications were imported into NVivo10 for qualitative analysis.

#### Analysis

Study data was collected and managed using REDCap. Quantitative data was exported to SPSS for analysis. Qualitative data from youth interviews and photos, and open-ended responses from questionnaires were managed using NVivo10.

Quantitative analysis of the organization and parent questionnaires was conducted separately. To achieve higher fidelity on the ratings of actionable solutions, only those rated as important or very important were selected as salient data points. We calculated frequencies and percentages of ratings of responses for each question.

For the qualitative analysis of questionnaire responses, each data point was treated separately. Open-ended responses were imported into NVivo software and coded by two team members (PY, JL) using a qualitative description approach (32). The analysis aimed at identifying proposed solutions and actions to promote the human rights of children with disabilities, with the purpose of informing a policy dialogue in childhood disabilities. These were grouped to represent each stakeholder group.

The analysis of the data generated with youth with disabilities was done separately. This data set was unique because it provided accounts of lived experience of disability and had the potential to greatly enrich “third party” accounts provided by parents and organizations representatives. Two members of the team (KS-T, GT) developed an analytical guide oriented by the study question addressed with youth participants, the scoping review and provisional analysis of earlier stakeholder dialogues. Table 1 presents the analytic questions developed to guide our qualitative analysis of the data generated with youth participants.

## Results

### Grassroots organizations

Forty-four federal grassroots organizations representing disability groups for children across Canada at federal and provincial levels completed the questionnaire. Twenty-five percent were federal organizations with a nation-wide mandate, 32% covered only Western provinces, 37% were from Central Canada, and 7% were from the Atlantic provinces. In addition, 20 of the over 200 smaller, local leisure-focused organizations that were listed in the Jooay App across 4 different provinces in Canada (Quebec, Alberta, Ontario, British Columbia) responded to the questionnaire. The responses from both groups (federal grassroots organizations and leisure organizations) were tested for differences in chosen priorities using *t*-tests. No differences were identified among the two groups (the only nearly significant difference was in the importance of family support; *p* = 0.052). The samples were then combined for analysis purposes for a total of 64 organizations (Table 2).

**Table 2:** Provincial distribution of participant organizations and parents.

	General	Leisure-specific	Total	Parents
Pan-Canadian	15	1	16	–
AB	7	3	10	9
BC	1	4	5	1
MB	0	0	0	1
NFLD	1	0	1	0
NS	1	0	1	1
ON	12	8	20	1
PEI	1	0	1	0
QC	6	4	10	12
Total	**44**	**20**	**64**	**25**

### Caregivers

The caregiver questionnaire was completed by 25 parents of children with diverse disabilities (children aged 3–27 years; mean 12.3 years) across multiple provinces (Table 2).

### Youth

Five youth (aged 16–21; 4 male, 1 female) participated in the study. Four participated in a subsequent interview (3 were audio-recorded and transcribed; 1 was summarized by the interviewer in field notes because the participant did not consent to audio-recording). One participant did not complete an interview but submitted reflections on their photos.

### Questionnaire results

The subset of participants that rated an issue as important or very important (a four or five on the 5-point scale) were included in further analysis. Those participants then selected solutions that addressed the issue at hand. The top-rated solutions are described in Table 3.

**Table 3:** Most important issues and solutions.

Issue	% orgs ranked as imp./very imp.	% parents ranked as imp./very imp.	Top solutions ranked for this issue
1. We need to identify and measure the physical, social, cultural, and economic barriers to full inclusion in order to develop policies, programs and interventions for children with disabilities	100	91	Measuring inclusion into communities as an indicator of success for interventions
			Using international human rights guidelines to guide standard of services
			Using international human rights guidelines to evaluate Canadian policies
2. Children with disabilities should be included in educational settings without discrimination from classmates and teachers	45	61	Reducing reliance on standardized tests of functioning to make decisions on education funding
			Encouraging the development of more discrete adaptive equipment for children with disabilities in classroom settings
			Developing programs to sensitize students to adaptive equipment used by children with disabilities in classrooms
3. Children with disabilities lack opportunities to participate equally in public life and in activities that are crucial for them to reach their full potential (e.g., leisure, community life, school activities, decision making	97	100	Ensuring that the voices, perspectives and experiences inform policy development and programming
			Facilitating participation in policymaking (e.g., consultations, hearings, public sessions and advocacy)
			Developing strategies that provide physical access to participate in public life
			Providing adequate levels of resources and services that support health and wellbeing
			Implementing positive approaches that encourage participation that reducing risk behaviours and increasing thriving
4. Lack of ownership and active engagement of children and youth with disabilities in decision-making related to them	77	76	Providing evidence-based strategies to support advocacy
			Developing programs to enable youth to develop self advocacy skills and to connect with the disability community
			Holding public consultations and policy dialogues with youth and evaluating such programs
			Raising awareness on rights-based approaches and the social model of disability
5. Strategies and policies promotion participation in leisure activities (e.g., sports and other recreational activities) for children with disabilities are lacking	91	88	Training physical education and community leisure providers on strategies to better address specific needs
			Developing and implementing policies promoting participation in leisure
			Providing early and cost-effective community-based interventions
6. Lack of integrated and adequate social, health, and educational services for children with disabilities, placing economic, psychological and medical burden of care from the state to the family (especially on women)	87	100	Encouraging family involvement with providers in decision-making
			Emphasizing an integrated approach across services that encourages greater coordination
			Facilitating and supporting community-based professional support services

#### Social inclusion

The first issue probed in the questionnaire began with the statement: “We need to identify and measure the physical, social, cultural, and economic barriers to full inclusion in order to develop policies, programs and interventions for children with disabilities”. All the organizations (100%) rated this issue as important or very important and most (91%) of parents rated it as such. Parents in particular highlighted the importance of this approach in the open comment box (“why is this issue important to you/your child?”):

*I have the expectation that my children, like all children, [would] be able to experience their world through whatever means they can accept in order for them to become the best versions of themselves. It would be unfair of me and others to make decisions for their outcome based on what they think is needed.—parent #3*

*Full inclusion is […] important because my child is a human being and deserves to be included.—parent #5*

*My son and our family need to be connected and supported in order to ensure he continues to grow into a well-adjusted adult, and that we are the best parents we can be for him.—parent #6*

*It is important to us that our child have community options for socialization and an ability to network, make friends and be part of society.—parent #11*

Organizations endorsed authentic engagement with families and community as part of the solution, as illustrated below:

*Sometimes, social inclusion by itself is not the best indicator of success. The quality of the social interactions is critical, and ensuring that social needs are identified and targeted for thoughtful intervention, building a community and team with the child and family, is important.—organization representative*

Eighty-five percent of organizations and 90% of parents endorsed the statement: “Measuring inclusion into communities as an indicator of success for interventions” is an important solution,

*Just because policy has been adequate in past does not mean it is keeping up with the societal changes and expectations … There is a disconnect between what has worked in past, what is available now and what could be made accessible.—parent #3*

Similarly, 72% of organizations and 86% of parents reported that “Using international human rights guidelines to guide standard of services” was an important solution. Lastly, 74% of organizations and 90% of parents thought “Using international human rights guidelines to evaluate Canadian policies” was an important or very important solution to addressing the human rights of children with disabilities, noting that using internationally developed policies and guidelines can be helpful in structuring policy directions:

*We believe the convention is a great guide for inclusion- in both creating and evaluating inclusion policies.—organization*

However, stakeholders also noted the importance of accounting for individual differences. Parents in particular perceived that there might be important challenges in implementing these treaties in a way that respects their child's unique and complex needs:

*While all these measures will be very useful, maybe try to also account for individuality, personality, variation among children.—parent #30*

#### Inclusive education

The second major issue highlighted on the questionnaire was that “Children with disabilities should be included in educational settings without discrimination from classmates and teachers”. Inclusive education was not a topic included in the research literature search that informed the questionnaire, yet, aspects related to inclusive education, and specifically to universal design in the education context, were brought up as closely connected to the notion of constructing a society that affirms human rights:

*Many technology aids can benefit all students—for example providing tablets for all students helps all students learn and ensures students with disabilities do not feel singled out. The more we can build UDL [Universal Design for Learning] into classrooms and teaching, the more all students can access the supports they need—organization representative*

*If we have integrated classrooms from the beginning of children's education, pre-k etc., I believe that it would become a normal/accepted practice for children and not even questioned. Look back to racial integration, [it becomes a] societal norm. We have constructed it this way.—parent #3*

Out of the 16 organizations and 11 parents that rated this issue as important or very important, three priority solutions were identified although organizations and parents ranked solutions differently. First, sixty-nine percent of organizations compared to 91% of parents endorsed “Developing programs to sensitize students to adaptive equipment used by children with disabilities in classrooms” as an important solution. “Reducing reliance of standardized tests of functioning to make decisions on educational funding,” was ranked by 75% of organizations as an important actionable solution while only 36% of parents thought this should be a priority. The third actionable solution (ranked highly by 69% of organizations and 55% of parents) involved “encouraging the development of more discrete adaptive equipment for children with disabilities in classroom settings”.

Stakeholders presented important considerations for implementation of these solutions, such as differentiating between equity and equality, addressing diversity of individual needs, and thinking beyond financial barriers to consider how social attitudes and expectations mediate social exclusion.

#### Participation in public life

The third issue within the questionnaire was that “children with disabilities lack opportunities to participate equally in public life and in activities that are crucial for them to reach their full potential (e.g., leisure, community life, school activities, decision-making)”.

Overall, there was a high level of agreement on the importance of this issue and the actionable solutions that were considered priorities in both groups. More solutions (*n* = 10) for this issue were identified in the research review than in the previous issues, therefore, we selected the top five of ten actionable solutions endorsed by both participant groups as important or very important. The majority of participants (100% of parents and 97% of parents) thought that “Ensuring that the voices, perspectives and experiences inform policy development and programming” was an important solution as was “Facilitating participation in policymaking (e.g., consultations, hearings, public sessions and advocacy)”. “Implementing positive approaches that encourage participation that reducing risk behaviours and increasing thriving” was endorsed by 88% of organizations and all the parents; “Developing strategies that provide physical access to participate in public life” was rated as important by all the organizations 94% of the parents; and finally, “Providing adequate levels of resources and services that support health and wellbeing” was rated as important by all the organizations and 94% of the parents.

Stakeholders elaborated on the accumulated challenges faced by parents to facilitate their child's participation in public life, and the fatigue and resignation that results:

*My experience with families is that by the time their child reaches mid-elementary school, they are tired of fighting with systems to ensure the rights of their children. If you are only 20% of the population, you are starting your fight as a minority and families express that fighting for equal treatment is exhausting. Even as 20% of the population, many of the battles are fought by one parent and 9 times out of 10, I witness them finally resigning themselves to accepting less.—organization*

#### Ownership and active engagement in decision-making

The fourth issue presented was “There is a lack of ownership and active engagement of children and youth with disabilities in decision-making related to them”. Out of the 6 actionable solutions presented, the top 4 endorsed by both groups (*n* = 27 organizations, 13 parents) are presented here.

There was a moderate level of agreement on the solutions addressing this issue. Eighty-one percent of organizations and 92% of parents said “Raising awareness on rights-based approaches and the social model of disability” was important. Ninety-three percent of organizations and 85% of parents thought that “Holding public consultations and policy dialogues with youth and evaluating such programs” was similarly important. “Developing programs to enable youth to develop self-advocacy skills and to connect with the disability community” was a priority solution to 89% of organizations and all parents while “Providing evidence-based strategies to support advocacy” was important to 70% of organizations and 85% of parents. Another key solution suggested by an organization was to *creat[e] connections between youth and role models/supports to encourage advocacy*.

Closely related to the theme of public participation, participants expressed a need to enact children's and youth's ability to advocate for themselves, and to engage at different levels of decision-making that affects them. For instance, one organization said: *We work with young men and women who want to advocate for themselves yet lack the resources or strategies to do so.* A parent (#15) added that, *Participation in decision-making promotes buy in and enhances self-esteem*. Barriers for children and youth to be more actively engaged in decision-making processes were presented not only as an issue related to lack of opportunities at the systems level, but also because parents do not feel capable of enacting their child's active engagement.

#### Leisure and community participation

The fifth issue was that “Strategies and policies promotion participation in leisure activities (e.g., sports and other recreational activities) for children with disabilities are lacking.”

Three of the four solutions from the literature were presented and rated by the 24 organizations and 16 parents that said this was an important issue to them. Ninety percent of organizations and all of the parents thought that “Providing early and cost-effective community-based interventions” was an important solution. Eighty percent of organizations and 93% of parents rated “Developing and implementing policies promoting participation in leisure for children with disabilities” as an important solution. And lastly, 93% of organizations and all the parents related “Training physical education and community leisure providers on strategies to better address specific needs” as important.

One organization said, *Recreation is so important for all children, and particularly for children with disabilities.* Yet, organizations and parents alike rated leisure as very important issue but commented on some key challenges to consider such as the need to tailor programs to individual needs and across the variety of special accommodations required for universal access. As one parent said, *Every disability is so different, you can't label what inclusion looks like, as it varies so so much*. Another key aspect highlighted was the dissonance between the perceived importance of leisure participation when compared to other unmet needs, as suggested by a parent (#3): *We are so behind the ball on simply having basic services available to families and children with disabilities, it seems like a frivolous ask to have* “*leisure activities*” *be a focus*.

Other key solutions by stakeholders included finding alternate methods for building capacity within community services providers, and developing infrastructure for exchanging information, obtaining equipment and providing the necessary supports:

*Identifying children with disabilities within communities so that providers can reach out with information and services. Providing equipment e.g., sport wheelchairs for those children.—organization*

*Inclusion*’ *as an art, science, and skill—it takes training and reflection and insight. [It takes] very deliberate and intentional thoughts and actions that are seldom including in any sector or profession.—organization*

#### Caregiver burden

The sixth and final issue presented was that “There is a lack of integrated and adequate social, health, and educational services for children with disabilities, placing economic, psychological and medical burden of care from the state to the family (especially on women).” Ninety-six percent of parents thought that this was very important while 58% of organizations are rated this issue at the level of importance to their organization. One parent wrote,

*This is my case, I am not working because [it] is very hard for me to be taking care of her 24 h [a day … ]. Sometimes I'm so tired, I'm upset, I'm angry because I'm tired. Medication, activity all the day, all the caring for her—mommy is friend, doctor, and I'm a human, it's not possible to have 24h of this during the whole life, this is impossible- life is very complicated.—parent #24*

The families' burden to navigate the systems, provide adequate supports for their child, and to eliminate systemic barriers for children and youth to be more actively engaged in decision-making processes were presented as one of the reasons not to be more active on promoting human rights:

*The lack of ownership isn't always due to lack of interest. I would love to be a better advocate for my children, to keep them involved in community activities and keep inclusion in the forefront of our lives. But I am sleep deprived, drowning in paperwork and burnt out already from this lifestyle. Meet us where we are. And we will gladly give you the information you are asking for.—parent #3*

The ratings of the 27 organizations and 17 parents on all four actionable solutions from the literature in the questionnaire are presented here. Ninety-three percent of organizations and 94% of parents rated the following three solutions as important: (1) Encouraging family involvement with providers in decision-making, (2) Emphasizing an integrated approach across services that encourages greater coordination, and (3) Facilitating and supporting community-based professional support services. Eighty-five percent of organizations and 88% of parents rated “Including homecare under the Canada Health Act” as important. The systemic barriers were highlighted in parent responses particularly, as illustrated in the following:

*I think it's really great in theory but much harder in practice. In some respects, I think families and parents do have voices with health care providers 1-on-1. But how you generalize that to actually have impact on service and on research and on health-providers. How you do it—system isn't really set up for this…—parent #4*

#### Youth voices

Overall, youth participants perceived human rights primarily as a matter of accessibility. More specifically, they reported that exercising their human rights was limited by factors summarized in the following four categories:

#### Inaccessible physical spaces

Many spaces in the community, including those that claim to be accessible, cannot be accessed independently by a youth in a wheelchair or with other physical limitations. Most photos taken by participants show how their human right to, *come and go* as they wish is constantly being violated. One youth, Ashir (all youth participant names are pseudonyms), commented that he wanted policy-makers to understand that, *disabled people are humans.* He appealed to the general public to *Make some ramps!* so he could get out of his apartment, go to a convenience store and buy something for himself, *just like everyone else*.

#### Transportation

Also related to accessibility and the right to come and go is the lack of accessible transportation options. Even though some buses were equipped with technology to accommodate wheelchairs, these ran infrequently, and youth felt that, overall, the drivers and passengers acted as though accommodating a person in a wheelchair was an inconvenience. Newer metro trains were described as lacking tie-down facilities which made youth feel extremely unsafe. Furthermore, scheduling adapted van services (an alternative to using regular buses or the metro) was experienced as a process that nearly always proved frustrating, causing participants to miss appointments or activities on a regular basis. One youth who had travelled by plane to visit family abroad found that the airline seemed ill-prepared to accommodate him and said it was exhausting to endure additional levels of security and multiple transfers from one wheelchair to another during the process of getting boarded.

#### Social stigma and intolerance

Lack of awareness about disability and the absence of other people with disabilities in many mainstream activities contributed to a sense of stigmatization and public intolerance. Several youth noted they were frequently stared at. Robert said, *It's kind of weird. It makes me feel bad. Sometimes, I tell them to back off*. This topic speaks to a youth's right to represent themselves and develop agency by increasing awareness of disability in the society at large.

#### Opportunities to participate in the community and be active

**Figure 2:**
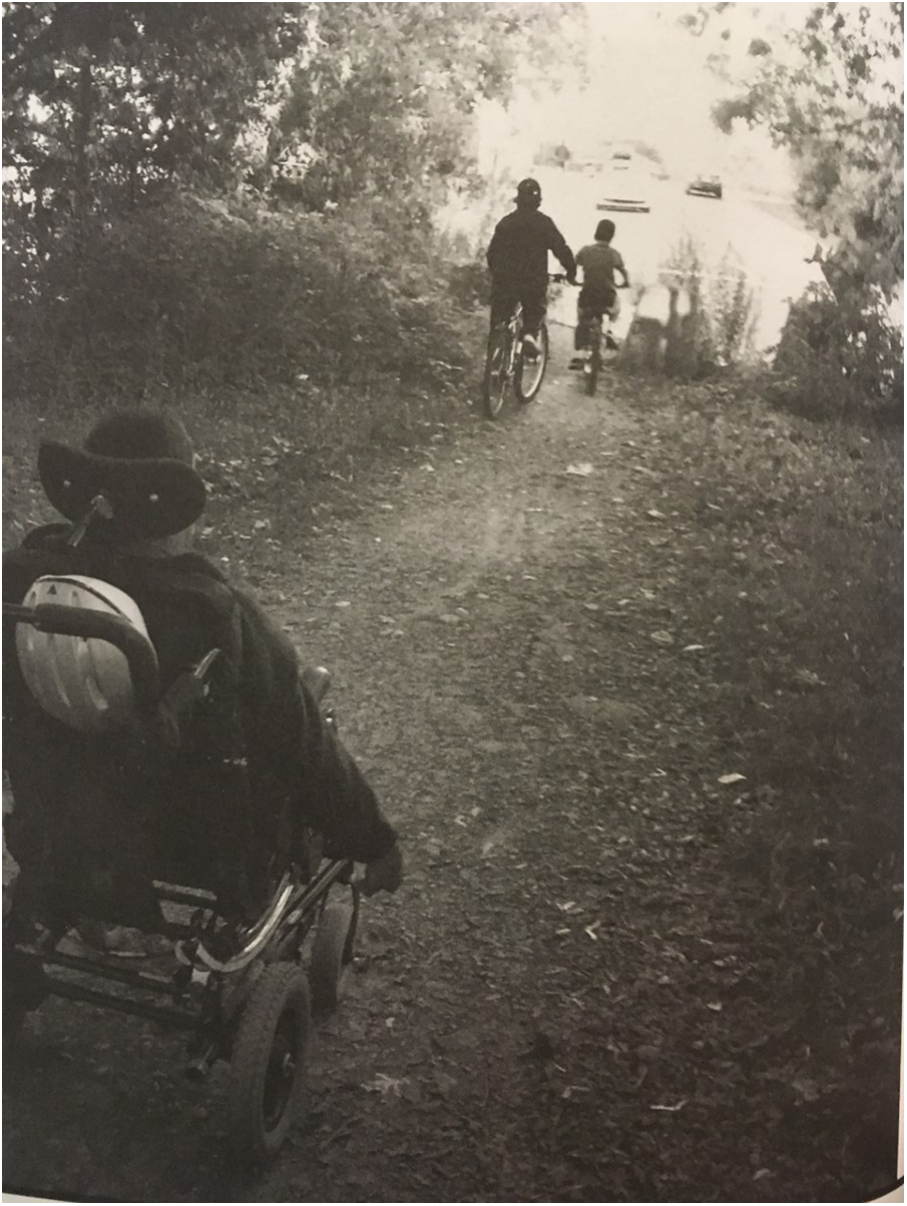
Participants picture illustrating his right to participate in the community.

**Figure 3:**
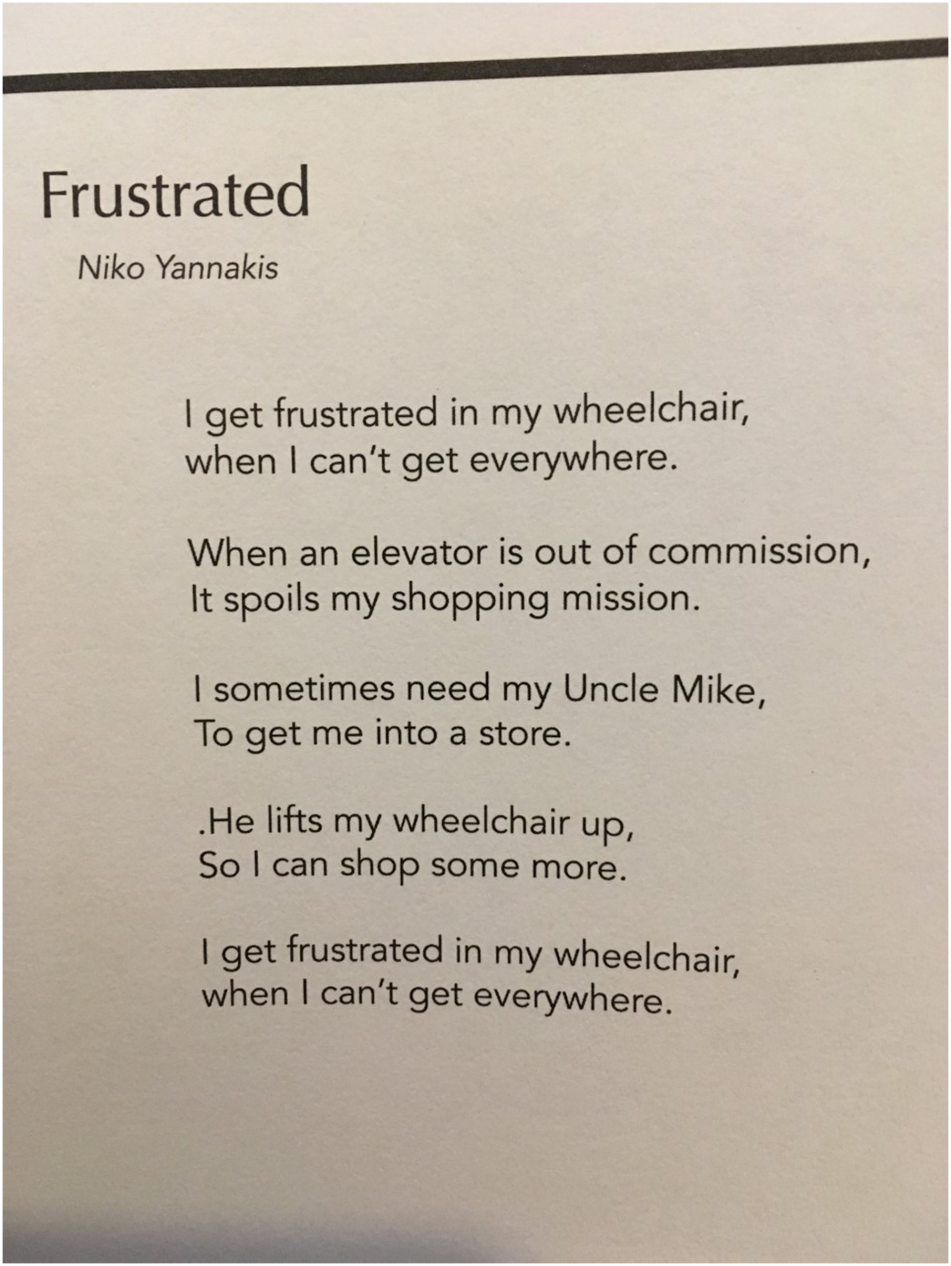
Participant's poem illustrating his right to participate in the community.

Disabling societal structures were highlighted as limiting opportunities for youth to engage in their communities, and participate in activities outside their home. For example, one youth described spending summers in the house playing video games instead of interacting and participating in the community. Youth shared ideas to address these situations: providing mobility equipment that would support their participation in outdoor activities, creating spaces and programs that were welcoming, and having people listening to their opinions on the matters that impact them. Bianca, a 17-year-old, expressed that participation was often restricted to interactions and activities were restricted to what happened in the school, and that this didn't allow for a broader understanding of what else she could do as a young person in relation to her human rights. Figure 2 presents a picture and Figure 3 presents a poem submitted by one of the participants as his view on how his rights are violated when he could not choose where to go (picture and real name used with permission).

**Figure 4:**
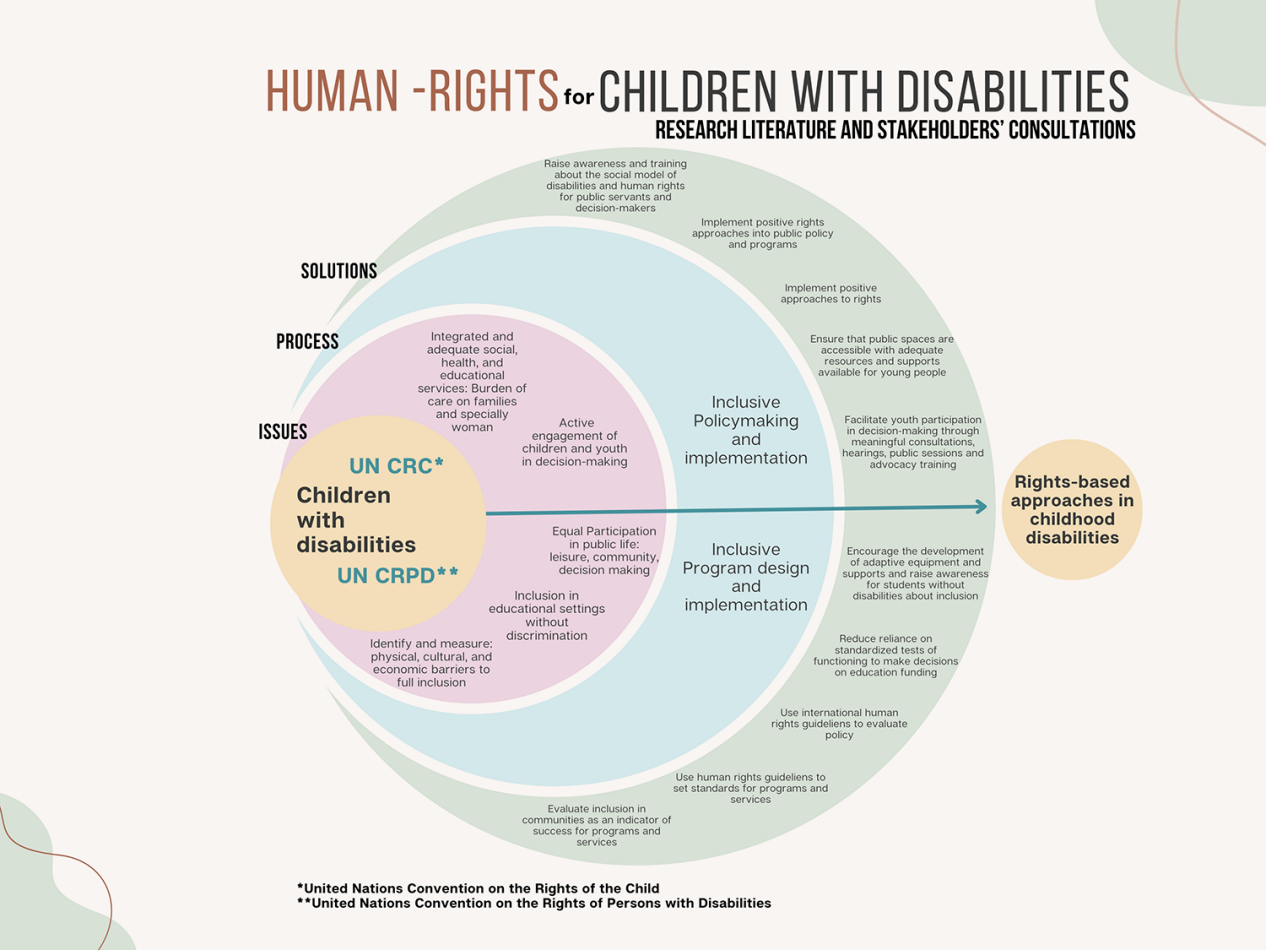
Conceptual map illustrating the issues identified in the research literature and prioritized through stakeholder consultations.

Figure 4 summarizes the issues, processes and solutions identified in the research review and prioritized through the stakeholders' consultations.

## Discussion

In this study, we identified the research literature on rights-based approaches in childhood disabilities and consulted with key stakeholders: Disability Persons Organizations (DPOs), Civil Society Organizations (CSOs), community-based organizations, parents of children with disabilities and youth with disabilities, on their perspectives of the importance of these approaches and the application to their daily lives. The study showed that DPOs as well as parents of children with disabilities agree overall on the importance of using rights-based approaches in policy and programs and in different sectors such as health and social services, community services and education. However, the approaches identified in the research literature are often too broad or high level (e.g., adopting human rights lenses into policymaking or using international human rights guidelines) to guide the standards of services making challenging for organizations, and especially for parents, to see the applicability of these rights in their daily lives. For specific issues pertaining to regulations and funding for organizations, parents and organizations did not agree on the level of importance.

Youth with disabilities and parents who participated in this study reported that they had limited exposure to the concepts related to human rights, and therefore, would think of rights as applied to aspects related to their daily routine. This finding resonates with previous research comparing parents' and youths' views. Parents of children with disabilities often report a lower quality of life of their child than children themselves (27). Parents also often value most aspects related relate to their child's care, including educational and health care needs, whereas children and youth value aspects that relate to their social participation and ability to make choices (27). This is an important perspective to take into consideration when developing programs and policies for children and youth. In enacting basic human rights principles such as “the best interests of the child” (CRC Article 9; CRPD Article 7) and the “right to voice their opinion” (CRC Article 13), governments, organizations and institutions should listen to the child, especially when the young person has communication restrictions, intellectual disabilities and other restrictions in their expression of their own opinions (33). Restrictions faced in this project in accessing the young people's opinions included limitations imposed by the school board members' beliefs on the ability of young people with severe disabilities to contribute to the project, the creativity and technical support required to allow for accessible communication, and parents, teachers, and technical staff willingness to mediate and facilitate communication. All of these barriers must be seen in the context of a social model of disability and should not impose limitations in the ability for young people to participate in different consultative processes (34). Appropriate systems must be put in place to address these needs and allow for equal participation in the development of public consultations leading to new policies and regulations in matters that are of interest to young people with disabilities, regardless of their accessibility needs (35, 36).

The lack of empirical research on the use of rights-based approaches to policymaking and service provision limits the research literature in informing policy and program development. In fact, organizations voiced that many of the rights-based approaches identified in the research were too vague to be applied to practice. Approaches that CSOs, advocacy organizations, and DPOs adopt were not referred to in the literature that we identified, reflecting the theoretical nature of this area. This opens the discussion for how approaching human rights in a more pragmatic manner. Some fields of study (e.g., critical disability studies and interdisciplinary human rights studies) have raised the issue of the lack of actionable recommendations in disability rights, and recommended ways to implement human rights in practice through adequate policies and programs (37).

This study also points to the importance of raising awareness and building capacity in human rights approaches among stakeholders. Participants with lived experiences need to understand the topic area of human rights to be able to meaningfully participate in the consultation process. The process itself must be framed in a way that resonates with their every-day lives. For instance, it is known that stakeholders' knowledge of the policy and regulation processes lead to better stakeholder engagement in policy development (38, 39). For meaningful consultations, researchers and decision-makers designing consultation processes must contextualize the information for stakeholders, and provide training or information sessions prior to the actual consultations, when the information they are being consulted on is beyond common knowledge or direct applications to daily life. The poor alignment between stakeholders' knowledge and needs and policymakers' or researchers' priorities must be addressed when developing participatory approaches to research and policy (40).Knowledge translation projects bridging the needs and knowledge level of stakeholders, including policymakers, regulators, legislators and others who might be involved in the application of rights-based approaches are necessary to improve the methods of consulting with stakeholders to inform research and policymaking (41).

We can also appreciate the importance of targeting specific groups for consultation by comparing the responses from organizations that are accustomed to working with the federal government in advocacy and decision-making processes and other organizations that offer programmatic inclusive leisure activities for children. While most organizations do not have a specific mandate to consider children's rights within disability rights, they understand the general frameworks of higher-level approaches such as a disability lenses or measuring policies according to their alignment with the UN conventions (42). The leisure community organizations however have a targeted clientele of children with disabilities, and therefore rated as important, the issues that are closer to the daily lives of children and families, and attributed great importance to approaches that relate to community inclusion and the promotion of leisure (43).

### Study limitations and future directions

The convenience sampling method presents some important limitations. First, organizations were sampled from lists of organizations that work with the Canadian government on an ongoing consultation process as well as those in a mobile app that lists inclusive leisure activities. They may not represent the full range of organizations that could have been identified through other sectors (e.g., education) or who do not interact with government. However, this is part of the design of the study and was not meant to be generalizable, but rather to shed light on the methods of using stakeholder consultation to evaluate the importance of research evidence, and to initiate a discussion on topics related to human rights identified by this community. For the young persons data point, the inclusion of only a small number of participants, and limited to those with physical limitations who were part of one specific classroom in a classroom for students with disabilities is also a limitation and one that had practical and ethical considerations. The use of multiple data collection methods is an important advance and strength of this study, as it allows for individual accommodations and the collection of data from young people who would not be able to participate in “traditional” data collection methods (e.g., interviews or focus groups). However, methods like photo elicitation while conducive to include non-verbal children and those who use augmentative and alternative communication, excludes children who are blind or have visual impairments. Many other groups such as deaf and hard of hearing youth and youth with intellectual disabilities were also not represented. Future studies should expand the inclusive and accessible methods and aim to include young people with diverse disabilities in consultations. The sample of parents who participated is limited to those with internet access, and who would have received the survey link through social media or the networks we connected with. Consideration for inclusion of families who may not have access to the internet, and are not part of networks, being likely the ones who have their rights to services and information violated, should be a priority in future research.

Another limitation of this study was the purposeful exclusion of rights-based approaches related to “education” in our research review. This decision was made in the beginning of the search as the preliminary search developed with multiple librarians yielded a large volume of articles related to education. For this reason, we decided that the topic of rights-based approaches in education deserved a dedicated review. Even though education was not included in this questionnaire, topics related to the importance of inclusive education were present. In this study, stakeholders identified inclusive education as important, but found, similarly, that the issue on access to assistive technology was too specific. These results can indicate that the notion of rights-based approaches is still in its infancy in the health field and has more actionable applications in education. Future studies should also incorporate stakeholders in the design phase of studies in the future. For example, parents, and organization representatives can help in the choice of keywords, and the initial analysis of data to continue guiding interpretation and development of questionnaires to yield more actionable solutions.

## Conclusion

Diverse groups of stakeholders perceive rights-based approaches in childhood disabilities as important. More education is needed to inform community-based organizations, parents, and youth about human rights and specifically children's and disability rights, and what they mean in practical terms in their daily lives. The academic community in diverse fields related to human rights, and those traditionally not adopting a human-rights framework such as health and rehabilitation, should consider incorporating this framework as part of the important outcomes of research studies. Furthermore, empirical studies that can test and verify the effectiveness of using certain rights-based approaches on the quality of life, well-being, community health, and other fundamental outcomes, are needed. Decision-makers must also understand and implement the concept of incorporating rights-based approaches in policy and program development that impact children with disabilities and their families.

## Data Availability

The raw data supporting the conclusions of this article will be made available by the authors, without undue reservation.

## References

[1] Sabatello M. The human rights of persons with intellectual disabilities: different but equal. Hum Rights Q. (2005) 27(2):737–49. 10.1353/hrq.2005.0025

[2] Shikako-Thomas K Law M. Policies supporting participation in leisure activities for children and youth with disabilities in Canada: from policy to play. Disabil Soc. (2015) 30(3):381–400. 10.1080/09687599.2015.1009001

[3] Shikako-Thomas K Shevell M. Promoting the human rights of children with neurologic conditions. Semin Pediatr Neurol. (2018) 27:53–61. 10.1016/j.spen.2018.03.007 30293590

[4] Convention on the Rights of the Child (1989).

[5] Convention on the Rights of Persons with Disabilities (2006).

[6] Brehaut JC Eva KW. Building theories of knowledge translation interventions: use the entire menu of constructs. Implement Sci. (2012) 7(1):1–10. 10.1186/1748-5908-7-114 PMC3520870 23173596

[7] Mitton C Adair CE McKenzie E Patten SB Waye Perry B. Knowledge transfer and exchange: review and synthesis of the literature. Milbank Q. (2007) 85(4):729–68. 10.1111/j.1468-0009.2007.00506.x 18070335 PMC2690353

[8] El-Jardali F Bou-Karroum L Ataya N El-Ghali HA Hammoud R. A retrospective health policy analysis of the development and implementation of the voluntary health insurance system in Lebanon: learning from failure. Soc Sci Med. (2014) 123:45–54. 10.1016/j.socscimed.2014.10.044 25462604

[9] Lavis JN Boyko JA Oxman AD Lewin S Fretheim A. SUPPORT tools for evidence-informed health policymaking (STP) 14: organising and using policy dialogues to support evidence-informed policymaking. Health Res Policy Syst. (2009) 7(1):1–8. 10.1186/1478-4505-7-S1-I1 20018104 PMC3271825

[10] Elsabbagh M Yusuf A Prasanna S Shikako-Thomas K Ruff CA Fehlings MG. Community engagement and knowledge translation: progress and challenge in autism research. Autism. (2014) 18(7):771–81. 10.1177/1362361314546561 25128332

[11] Fishbein DH Ridenour TA Stahl M Sussman S. The full translational spectrum of prevention science: facilitating the transfer of knowledge to practices and policies that prevent behavioral health problems. Transl Behav Med. (2016) 6(1):5–16. 10.1007/s13142-015-0376-2 27012249 PMC4807200

[12] Welch VA Petticrew M O'Neill J Waters E Armstrong R Bhutta ZA Health equity: evidence synthesis and knowledge translation methods. Syst Rev. (2013) 2(1):1–10. 10.1186/2046-4053-2-43 23799964 PMC3702469

[13] Fox DM. Evidence of evidence-based health policy: the politics of systematic reviews in coverage decisions. Health Aff. (2005) 24(1):114–22. 10.1377/hlthaff.24.1.114 15647221

[14] Oliver K Lorenc T Innvær S. New directions in evidence-based policy research: a critical analysis of the literature. Health Res Policy Syst. (2014) 12(1):1–11. 10.1186/1478-4505-12-34 25023520 PMC4107868

[15] Oliver K Innvar S Lorenc T Woodman J Thomas J. A systematic review of barriers to and facilitators of the use of evidence by policymakers. BMC Health Serv Res. (2014) 14(1):1–12. 10.1186/1472-6963-14-2 24383766 PMC3909454

[16] Contandriopoulos D Lemire M Denis JL Tremblay E. Knowledge exchange processes in organizations and policy arenas: a narrative systematic review of the literature. Milbank Q. (2010) 88(4):444–83. 10.1111/j.1468-0009.2010.00608.x 21166865 PMC3037172

[17] Liverani M Hawkins B Parkhurst JO. Political and institutional influences on the use of evidence in public health policy. A systematic review. PLoS One. (2013) 8(10):e77404. 10.1371/journal.pone.0077404 24204823 PMC3813708

[18] Orem JN Mafigiri DK Marchal B Ssengooba F Macq J Criel B. Research, evidence and policymaking: the perspectives of policy actors on improving uptake of evidence in health policy development and implementation in Uganda. BMC Public Health. (2012) 12(1):1–16. 10.1186/1471-2458-12-1 22316003 PMC3305540

[19] Cairney P Oliver K. Evidence-based policymaking is not like evidence-based medicine, so how far should you go to bridge the divide between evidence and policy? Health Res Policy Syst. (2017) 15(1):1–11. 10.1186/s12961-017-0192-x 28446185 PMC5407004

[20] Camden C Shikako-Thomas K Nguyen T Graham E Thomas A Sprung J Engaging stakeholders in rehabilitation research: a scoping review of strategies used in partnerships and evaluation of impacts. Disabil Rehabil. (2015) 37(15):1390–400. 10.3109/09638288.2014.963705 25243763

[21] Tricco AC Zarin W Rios P Nincic V Khan PA Ghassemi M Engaging policy-makers, health system managers, and policy analysts in the knowledge synthesis process: a scoping review. Implement Sci. (2018) 13(1):1–19. 10.1186/s13012-017-0699-0 29433543 PMC5809959

[22] Fudge N Wolfe CD McKevitt C. Assessing the promise of user involvement in health service development: ethnographic study. Br Med J. (2008) 336(7639):313–7. 10.1136/bmj.39456.552257.BE 18230646 PMC2234509

[23] Deshefy-Longhi T Sullivan–Bolyai S Dixon J. Data collection order: a primer. South Online J Nurs Res. (2009) 9(3):6. PMID: . 20671807 PMC2911037

[24] Schoonenboom J Johnson RB. How to construct a mixed methods research design. KZfSS Kölner Zeitschrift für Soziologie und Sozialpsychologie. (2017) 69(2):107–31. 10.1007/s11577-017-0454-1 28989188 PMC5602001

[25] Boyko JA Lavis JN Dobbins M. Deliberative dialogues as a strategy for system-level knowledge translation and exchange. Healthc Policy. (2014) 9(4):122. PMID: . 24973488 PMC4749889

[26] Teachman G Mistry B Gibson BE. Doing Qualitative Research with People Who Have Communication Impairments. London: SAGE Research, Ltd. (2014). 10.4135/978144627305013514660

[27] Teachman G Gibson BE. Children and youth with disabilities: innovative methods for single qualitative interviews. Qual Health Res. (2013) 23(2):264–74. 10.1177/1049732312468063 23208200

[28] Croghan R Griffin C Hunter J Phoenix A. Young people’s constructions of self: notes on the use and analysis of the photo-elicitation methods. Int J Soc Res Methodol. (2008) 11(4):345–56. 10.1080/13645570701605707

[29] Epstein I Stevens B McKeever P Baruchel S. Photo elicitation interview (PEI): using photos to elicit children’s perspectives. Int J Qual Methods. (2006) 5(3):1–11. 10.1177/160940690600500301

[30] Sandelowski M. Whatever happened to qualitative description? Res Nurs Health. (2000) 23(4):334–40. 10.1002/1098-240X(200008)23:4<334::AID-NUR9>3.0.CO;2-G 10940958

[31] Payot A Barrington KJ. The quality of life of young children and infants with chronic medical problems: review of the literature. Curr Probl Pediatr Adolesc Health Care. (2011) 41(4):91–101. 10.1016/j.cppeds.2010.10.008 21440223

[32] Shikako-Thomas K Majnemer A Law M Lach L. Determinants of participation in leisure activities among adolescents with cerebral palsy. Res Dev Disabil. (2013) 34(9):2621–34. 10.1016/j.ridd.2013.05.013 23751302

[33] Cavet J Sloper P. Participation of disabled children in individual decisions about their lives and in public decisions about service development. Child Soc. (2004) 18(4):278–90. 10.1002/chi.803

[34] Lang R Kett M Groce N Trani JF. Implementing the United Nations Convention on the rights of persons with disabilities: principles, implications, practice and limitations. Alter. (2011) 5(3):206–20. 10.1016/j.alter.2011.02.004

[35] Baker P Moon NW. Policy development and access to wireless technologies for people with disabilities: results of policy Delphi research. Univers Access Inf Soc. (2010) 9(3):227–37. 10.1007/s10209-009-0170-3

[36] Seddoh A Akor SA. Policy initiation and political levers in health policy: lessons from Ghana’s health insurance. BMC Public Health (2012) 12 (Suppl 1):S10. 10.1186/1471-2458-12-S1-S10 22992292 PMC3381692

[37] Fretheim A Schünemann HJ Oxman AD. Improving the use of research evidence in guideline development: 3. Group composition and consultation process. Health Res Policy Syst. (2006) 4(1):1–6. 10.1186/1478-4505-4-1 17134482 PMC1702349

[38] Graham ID Logan J Harrison MB Straus SE Tetroe J Caswell W Lost in knowledge translation: time for a map? J Contin Educ Health Prof. (2006) 26(1):13–24. 10.1002/chp.47 16557505

[39] Kothari A Boyko JA Conklin J Stolee P Sibbald SL. Communities of practice for supporting health systems change: a missed opportunity. Health Res Policy Syst. (2015) 13(1):1–9. 10.1186/1478-4505-13-1 26208500 PMC4515005

[40] Chadha E. Running on empty: the not so special status of paratransit services in Ontario. Windsor Rev Legal Soc Issues. (2005) 20:1.

[41] Rosenthal E Bauer E Hayden MF Holley A. Implementing the right to community integration for children with disabilities in Russia: a human rights framework for international action. Health Hum Rights. (1999) 4:82. PMID: . 10438556

[42] Russell F. The expectations of parents of disabled children. Br J Spec Educ. (2003) 30(3):144–9. 10.1111/1467-8527.00300

[43] Bickenbach J. Universally design social policy: when disability disappears? Disabil Rehabil. (2014) 36(16):1320–7. 10.3109/09638288.2014.932447 24954389

